# New genus of the family Laophontidae T. Scott, 1905 (Copepoda, Harpacticoida) from Hupo on the eastern coast of Korea

**DOI:** 10.3897/BDJ.12.e108106

**Published:** 2024-02-12

**Authors:** Je Hyeok Oh, Dongsung Kim, Wonchoel Lee

**Affiliations:** 1 Korea Institute of Ocean Science and Technology, Busan, South Korea Korea Institute of Ocean Science and Technology Busan South Korea; 2 Hanyang University, Laboratory of Biodiversity, Seoul, South Korea Hanyang University, Laboratory of Biodiversity Seoul South Korea

**Keywords:** Harpacticoid copepods, meiofauna, taxonomy, *Strictlaophonte*, East Sea, subtidal zone

## Abstract

**Background:**

The Laophontidae is a very large and diverse family containing more than 320 species and 74 genera in the Harpacticoida. According to records published until recently, 25 species of 12 genera of Laophontidae were reported to appear in Korean waters. The most common laophontid species in Korean waters is *Paralaophontecongenera* (Sars, 1908). During research on the meiobenthic community in the subtidal zone near the Korean coast in the East Sea, we found an undescribed genus of the Laophontidae family. The character traits of this undescribed specimen of the Laophontidae family do not match any existing genus.

**New information:**

Herein, a new genus of the interstitial marine benthic copepods family Laophontidae T. Scott, 1905 is described from the subtidal zone near Hupo Harbour on the east coast of Korea. This genus was named *Strictlaophonte* gen. nov. and has been classified into the family Laophontidae. This was based on the presence of seven segmented female antennules, reduced antennary exopod, first endopodal segments having no inner setae from the second leg to the fourth leg and P5 with a distinct exopod that is not fused at the basis. The distinguishing features of *Strictlaophonte* gen. nov. are P5 exopod having only four setae, the P1 exopod having two segments and the antenna exopod having four setae. In particular, this new genus has unique characteristics in that the caudal rami are very tightly attached to each other.

## Introduction

The family Laophontidae T. Scott, 1905 is essentially marine, free-living and benthic, mainly inhabiting the intertidal zone or shallow subtidal habitats and is commonly found amongst algae and seagrass (Huys et al. 1996, Boxshall and Halsey 2004, Kaymak and Karaytuğ 2014). Additionally, the Laophontidae is a very large and diverse family containing more than 320 species and 74 genera in the Harpacticoida (Huys and Lee 2018, Kim et al. 2020).

The type species, *Laophontecornuta*, was published by Philippi (1840) and, since then, new genera and species have been classified by many researchers. In addition, genera and species that were unnaturally and polyphyletically established during the long history of classification in the past have recently been re-established as phylogenetically convincing genera and species (Huys 2008, Kaymak and Karaytuğ 2014). According to records published until recently, 25 species in 12 genera of the Laophontidae were reported to appear in Korean waters (Huys and Lee 2018) and Lee et al. (2012) referred *Paralaophontecongenera* (Sars, 1908) as the most common laophontid species in the Korea.

During research on the meiobenthic community in the subtidal zone near the Korean coast in the East Sea, we found an undescribed genus of the family Laophontidae. The subtidal sediments adjacent to Hupo are predominantly sandy and algae and seagrass are abundant in the water. Sandy bottoms are generally considered an unfavourable environment for meiofauna compared to mud, although copepods can successfully inhabit sandy sediments (Boxshall and Halsey 2004). Additionally, most harpacticoid copepods have interstitial forms that can colonise the mesopsammic environment because of their cylindrical body shapes with reduced setal armature of thoracic legs (Noodt 1971, Martinez Arbizu and Moura 1994, Huys and Conroy-Dalton 2006, Corgosinho and Schizas 2012). The family Laophontidae contains a variety of genera adapted to habitat types (Gheerardyn et al. 2007). Accordingly, as a result of taxonomic investigation, we here erect a new genus, based on newly-collected specimens from the subtidal zone near Hupo Port located on the east coast of Korea.

## Materials and methods

**Figure 1.**
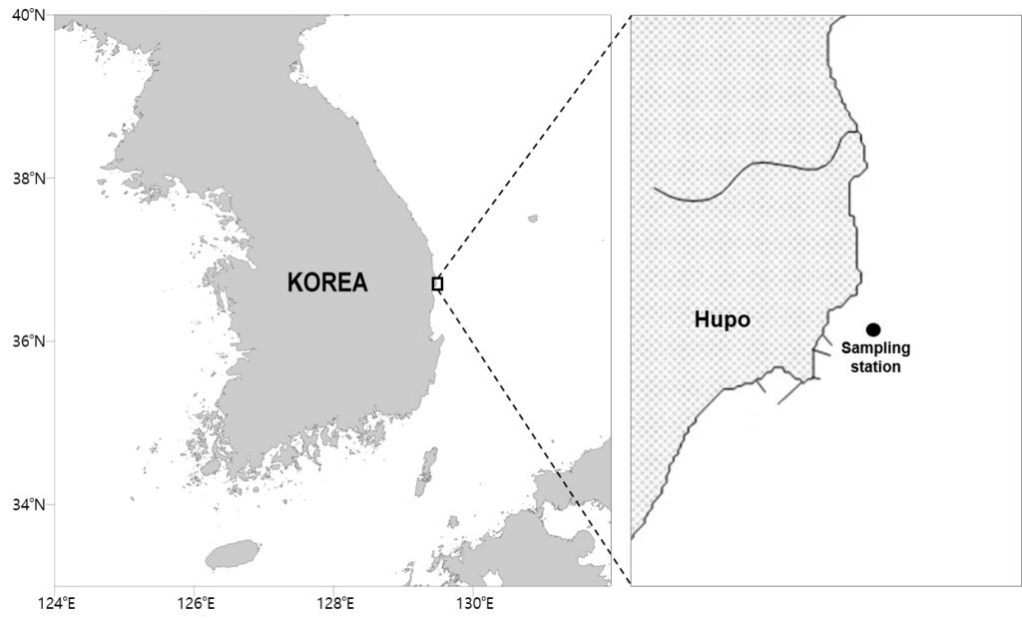
The location of the sampling station on the east coast of Korea.

Samples were collected from the subtidal zone at Hupo on the east coast of Korea (Fig. 1). Sandy sediment was sampled by grabbing (surface area 0.1 m^2^) on board and were then sub-sampled using acrylic corers (diameter 3.57 cm). Collected samples were then fixed in 5% neutral formalin for 1 h and transferred to 70% ethanol for preservation. After the samples were brought to the laboratory, fauna and sediment were separated using Ludox, a colloidal silica solution with a specific gravity very close to that of the fauna and a centrifuge (Burgess 2001), before copepods were sorted from the organic material. Specimens were dissected in lactic acid and then mounted on slides. All drawings were prepared using a drawing tube mounted on an Olympus BX51 differential interference contrast microscope. The description of terminology was adopted from Huys et al. (1996). Abbreviations used in the text are: A1, antennule; A2, antenna; ae, aesthetasc; exp, exopod; enp, endopod; P1–P4, first to fourth thoracic legs; P5–P6, fifth to sixth legs; exp(enp)-1 (-2, -3) denotes the proximal (middle, distal) segment of a ramus. Type specimens of this new species are deposited at the Marine Biodiversity Institute of Korea (MABIK). The scale bars in the figures are in micrometres.

## Taxon treatments

### Strictlaophonte

Oh, Kim & Lee gen. nov.

E07DE873-510C-5050-A25B-476C5D1CB9FC

8F853C9B-209B-4046-8E13-C8EA3EE618D7

Strictlaophonte hupoensis Oh, Kim & Lee, 2024. Type species.

#### Diagnosis

Laophontidae. Body slender, sub-cylindrical; urosome narrower than prosome and cephalothorax with integument coverted in pitted marks. Rostrum triangular and completely fused to cephalothorax. Genital field with pair of P6 widely separated and each leg represented by 2 setae. Sexual dimorphism in antennules, P3, P5, P6 and genital segmentation. Anal operculum convex, anal opening bordered by setular rows. Caudal rami with right and left rami including terminal seta tightly attached to each other. Antennule 7-segmented in female, 8-segmented in male; second segment with horn-shaped integumental protuberance. Antennary exopod with 4 setae. P1 exopod small, 2-segmented, not exceeding middle of Enp-1. P2-P4 all endopods 2-segmented and exopod 3-segmented, except male P3 endopod 3-segmented; enp-1 without inner and outer setae. Male P3 Enp-2 with 1 strong inner apophysis. Female P5 with 5 setae on endopod and 4 on exopod; endopodal lobe exceeding distal margin of small exopod; male endopodal and exopodal lobes vestigial, the former with 2 and the latter 3 setae. Type species monotypic.

#### Etymology

The generic name is derived from the Latin 'strictus', meaning 'close' or 'tight', referring to the tightly attached caudal rami (including main setae on both rami).

### Strictlaophonte hupoensis

Oh, Kim & Lee sp. nov.

6B9CC2E5-54B0-5952-87DD-1EF68E190655

B2D1FBBC-B2A0-4EF7-BD10-5132A7AB8BD8

#### Materials

- **Type status:** Holotype. **Occurrence:** individualCount: 1; sex: female; lifeStage: adult; preparations: dissected on 1 slides; occurrenceID: B19B8AB6-A78E-53FB-86BC-29A5719B76CF; **Taxon:** scientificName: *Strictlaophontehupoensis*; kingdom: Animalia; phylum: Arthropoda; class: Copepoda; order: Harpacticoida; family: Laophontidae; genus: Strictlaophonte; specificEpithet: *hupoensis*; taxonRank: species; scientificNameAuthorship: Oh, Kim & Lee; **Location:** higherGeography: East Asia; waterBody: East Sea; country: Korea; countryCode: KR; stateProvince: Gyeongsangbuk-do; county: Uljin-gun; municipality: Hupo-myeon; locality: Subtidal zone 2.2 km NE from Hupo Port; verbatimDepth: 15.3 m; verbatimLatitude: 36°41'31.4''N; verbatimLongitude: 129°28'51.60''E; **Identification:** identifiedBy: Oh, Kim & Lee; dateIdentified: 2024; **Event:** samplingProtocol: Smith-McIntyre grab; eventDate: 27/07/2010; **Record Level:** language: en; basisOfRecord: PreservedSpecimen
- **Type status:** Paratype. **Occurrence:** individualCount: 1; sex: female; lifeStage: adult; preparations: dissected on 1 slides; occurrenceID: 78E3C7A0-0725-5C65-A026-CAF492253676; **Taxon:** scientificName: *Strictlaophontehupoensis*; kingdom: Animalia; phylum: Arthropoda; class: Copepoda; order: Harpacticoida; family: Laophontidae; genus: Strictlaophonte; specificEpithet: *hupoensis*; taxonRank: species; scientificNameAuthorship: Oh, Kim & Lee; **Location:** higherGeography: East Asia; waterBody: East Sea; country: Korea; countryCode: KR; stateProvince: Gyeongsangbuk-do; county: Uljin-gun; municipality: Hupo-myeon; locality: Subtidal zone 2.2 km NE from Hupo Port; verbatimDepth: 15.3 m; verbatimLatitude: 36°41'31.45''N; verbatimLongitude: 129°28'51.60''E; **Identification:** identifiedBy: Oh, Kim & Lee; dateIdentified: 2024; **Event:** samplingProtocol: Smith-McIntyre grab; eventDate: 27/07/2010; **Record Level:** language: en; basisOfRecord: PreservedSpecimen
- **Type status:** Paratype. **Occurrence:** individualCount: 2; sex: male; lifeStage: adult; preparations: dissected on 1 slides; occurrenceID: 2DD619EA-B545-5EE6-A705-3E9140D216CB; **Taxon:** scientificName: *Strictlaophontehupoensis*; kingdom: Animalia; phylum: Arthropoda; class: Copepoda; order: Harpacticoida; family: Laophontidae; genus: Strictlaophonte; specificEpithet: *hupoensis*; taxonRank: species; scientificNameAuthorship: Oh, Kim & Lee; **Location:** higherGeography: East Asia; waterBody: East Sea; country: Korea; countryCode: KR; stateProvince: Gyeongsangbuk-do; county: Uljin-gun; municipality: Hupo-myeon; locality: Subtidal zone 2.2 km NE from Hupo Port; verbatimDepth: 15.3 m; verbatimLatitude: 36°41'31.45''N; verbatimLongitude: 129°28'51.60''E; **Identification:** identifiedBy: Oh, Kim & Lee; dateIdentified: 2024; **Event:** samplingProtocol: Smith-McIntyre grab; eventDate: 27/07/2010; **Record Level:** language: en; basisOfRecord: PreservedSpecimen
- **Type status:** Paratype. **Occurrence:** individualCount: 4; sex: female; lifeStage: adult; preparations: preserved in 90% ethanol; occurrenceID: D348FC87-300E-5D4D-B58E-0D57C1A66EE2; **Taxon:** scientificName: *Strictlaophontehupoensis*; kingdom: Animalia; phylum: Arthropoda; class: Copepoda; order: Harpacticoida; family: Laophontidae; genus: Strictlaophonte; specificEpithet: *hupoensis*; taxonRank: species; scientificNameAuthorship: Oh, Kim & Lee; **Location:** higherGeography: East Asia; waterBody: East Sea; country: Korea; countryCode: KR; stateProvince: Gyeongsangbuk-do; county: Uljin-gun; municipality: Hupo-myeon; locality: Subtidal zone 2.2 km NE from Hupo Port; verbatimDepth: 15.3 m; verbatimLatitude: 36°41'31.45''N; verbatimLongitude: 129°28'51.60''E; **Identification:** identifiedBy: Oh, Kim & Lee; dateIdentified: 2024; **Event:** samplingProtocol: Smith-McIntyre grab; eventDate: 27/07/2010; **Record Level:** language: en; basisOfRecord: PreservedSpecimen
- **Type status:** Paratype. **Occurrence:** individualCount: 3; sex: male; lifeStage: adult; preparations: preserved in 90% ethanol; occurrenceID: A5FBEE81-C443-599C-B4C6-C279CF29A350; **Taxon:** scientificName: *Strictlaophontehupoensis*; kingdom: Animalia; phylum: Arthropoda; class: Copepoda; order: Harpacticoida; family: Laophontidae; genus: Strictlaophonte; specificEpithet: *hupoensis*; taxonRank: species; scientificNameAuthorship: Oh, Kim & Lee; **Location:** higherGeography: East Asia; waterBody: East Sea; country: Korea; countryCode: KR; stateProvince: Gyeongsangbuk-do; county: Uljin-gun; municipality: Hupo-myeon; locality: Subtidal zone 2.2 km NE from Hupo Port; verbatimDepth: 15.3 m; verbatimLatitude: 36°41'31.45''N; verbatimLongitude: 129°28'51.60''E; **Identification:** identifiedBy: Oh, Kim & Lee; dateIdentified: 2024; **Event:** samplingProtocol: Smith-McIntyre grab; eventDate: 27/07/2010; **Record Level:** basisOfRecord: PreservedSpecimen

#### Description

**Figure 2.**
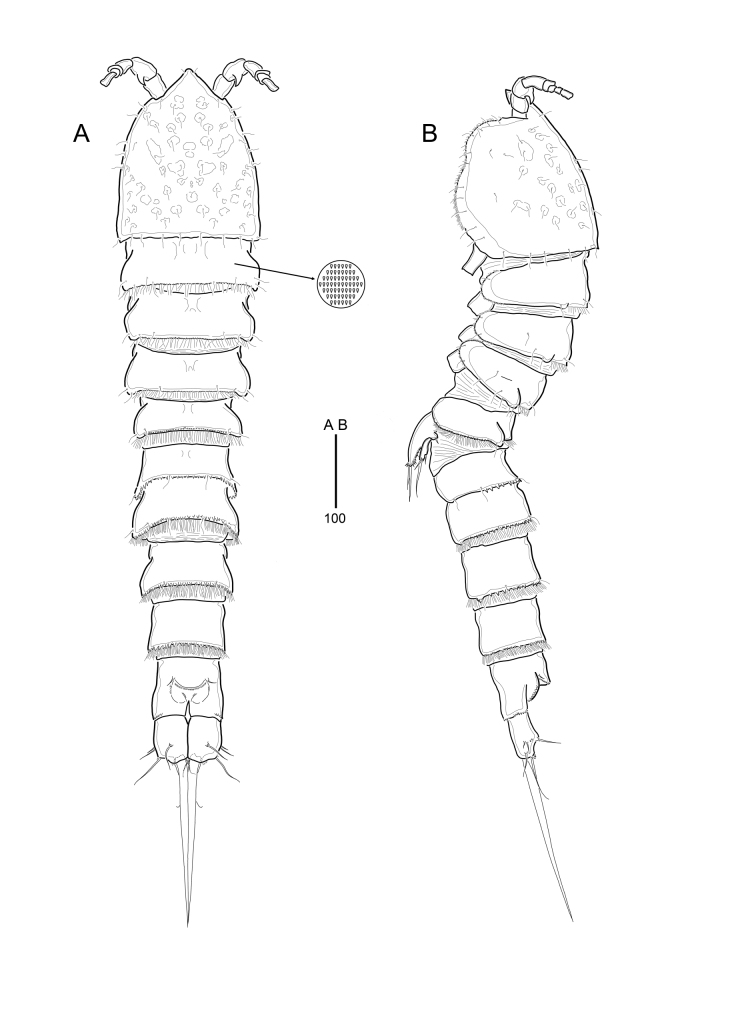
*Strictlaophontehupoensis* gen. nov., sp. nov., female; **A** habitus, dorsal; **B** habitus, lateral.

**Female**: Body slender. Body length 1226 μm (range 1158 - 1292 μm, measured from anterior margin of rostrum to posterior margin of caudal rami). Largest width measured at posterior margin of cephalic shield: 185 μm. Entire body surface bumpy. Urosome narrower than prosome (Fig. 2A and B).

Cephalothorax with posterior margin fringed with small spinules and pattern of sensillae; integument coverted in pitted marks of various shapes and sizes. Rostrum triangular (Fig. 2A), completely fused to cephalothorax and with pair of sensilla near apex. All somites covered with tiny invisible, cuticle-patterned integument. There are postero-lateral protrusions on the dorsal surface of P2-P5 bearing-somites. Pleural areas well-developed and rounded, with lobate posterolateral angles. Several sensilla are present in all somites, except for the penultimate somite as in Fig. 2A, B. Pedigerous somites without hyaline frills, posterior margins fringed with small setules.

**Figure 3.**
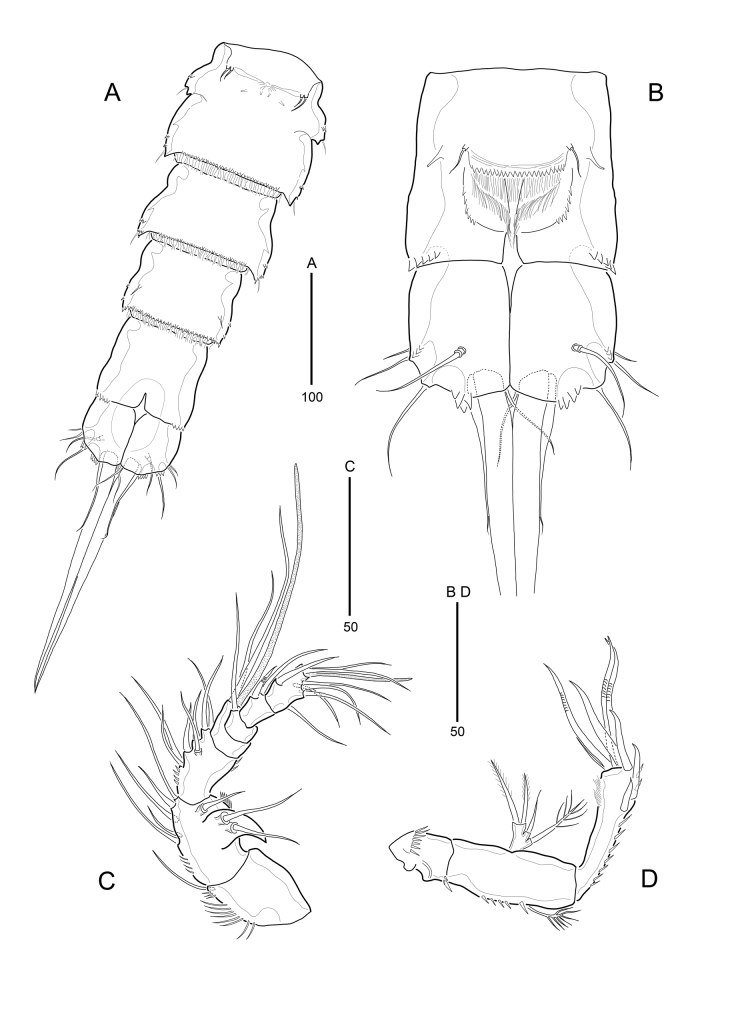
*Strictlaophontehupoensis* gen. nov., sp. nov., female; **A** urosome, ventral; **B** anal segment and caudal rami, dorsal; **C** antennule; **D** antenna.

Urosome (Fig. 2A, B and Fig. 3A) gradually tapering posteriorly, five-segmented, comprising P5-bearing somite, genital double-somite, two free abdominal somites, and anal somite. Urosome without hyaline frills, with posterior margin fringed with setules except for anal somites.

Genital double-somite (Fig. 2A, B and Fig. 3A) with transverse surface ridge dorsally and laterally, indicating original segmentation and completely fused ventrally. Genital field (Fig. 3A) located near anterior margin with small-sized copulatory pore located in median depression. Pair of P6 widely separated, in genital field, each leg represented by small protuberence bearing 2 bare setae; outer seta slightly longer than inner seta.

Anal somite (Fig. 3B) with pair of dorsal tube-pores. Operculum semi-weakly convex, with serrate postrior margin and pair of sensilla on both sides. Anal opening triradiate, bordered by well-developed and serrate frill bearing setular rows.

Caudal rami (Fig. 3A and B) cylindrical, 1.5 times longer than wide; right and left rami including seta V tightly attached to each other; each ramus with 7 naked setae: seta I bare and shortest, seta II shorter than seta III, seta IV much shorter than seta V; seta V longest and very thick, shorter than urosome, 235 μm long; seta VI bare and small, dorsal seta VII tri-articulate at base, slightly shorter than seta III; additional spinular ornamentation present around distal margins.

Antennule (Fig. 3C) 7-segmented; fifth segment shortest, second segment longest. Segment 1 with spinular row at inner margin; with a long spinule at distal anterior margin. Second segment with horn-shaped integumental protuberance on outer middle margin; with row of small spinules on inner and outer margins. Segment 3 with row of small spinules on inner margin. Armature formula: 1-[1], 2-[8], 3-[7], 4-[1 + (1 + ae)], 5-[1], 6-[2], 7-[6 + acrothek]. Apical acrothek consisting of small aesthetasc fused basally to 2 bare setae.

Antenna (Fig. 3D) 3-segmented, comprising coxa, allobasis and free 1-segmented endopod. Coxa small, with two rows of spinules. Allobasis elongated; row of spinules on the side; without distinct surface sutures marking original segmentation, with 1 abexopodal seta near middle. Exopod small, with 2 pinnate setae apically, 1 naked seta and 1 plumose seta laterally. Endopod about as long as allobasis. Lateral armature consisting of 2 strong spines; apical armature consisting of 2 strong spines, and 3 geniculate setae (1 geniculate seta fused basally to additional short seta). Endopod with row of long spinules laterally and with frills along the outer edge surface.

**Figure 4.**
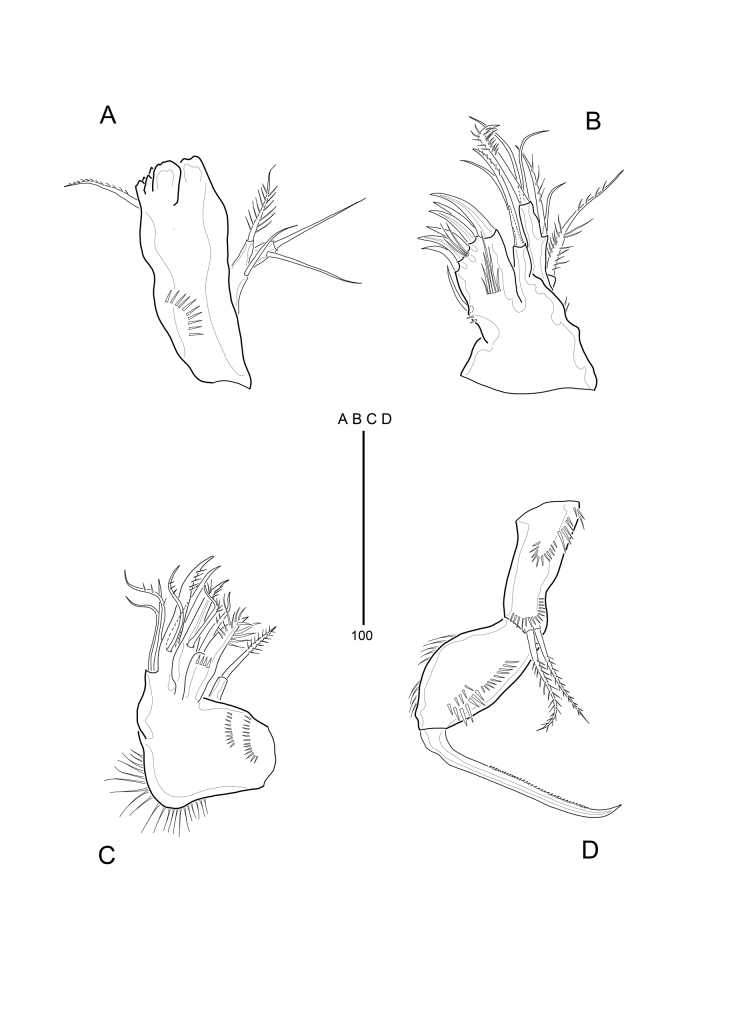
*Strictlaophontehupoensis* gen. nov., sp. nov., female; **A** mandible; **B** maxillule; **C** maxilla; **D** maxilliped.

Mandible (Fig. 4A) with well-developed gnathobase, bearing several multicuspidate teeth around distal margin and 1 long pinnate seta at distal corner. Coxa with row of spinules proximally. Mandibular palp 2-segmented, proximal segment (basis) with 1 plumose seta and 1 naked seta, distal segment (endopod) with 2 naked setae.

Maxillule (Fig. 4B). Praecoxa with little spinules around outer margin; arthrite strongly developed, with 1 naked seta on dorsal surface and 6 spines/setae around distal margin; with row of various-sized spinules on anterior surface and row of long spinules on anterior surface. Coxa with cylindrical endite bearing 1 naked seta and 1 pinnate spine. Basis with cylindrical endite bearing 2 naked setae, and 1 strong, curved, pinnate spine; on distall margin, endopod nearly incorporated into basis, forming small peduncle with 1 naked seta and 1 pinnate seta. Exopod 1-segmented, with 1 long pinnate seta.

Maxilla (Fig. 4C). Syncoxa with 3 endites; with two rows of spinules on anterior surface and row of setules on outer margin as figured. Proximal endite small and with 1 strong, pinnate spine. Middle endite with 2 pinnate setae, of which proximal one well-developed and fused basally to endite. Distal endite drawn out into claw-like protrusion bearing 1 distally unipinnate seta. Allobasis produced into strong pinnate claw, with 1 naked seta posteriorly and 1 slender pinnate anterior seta. Endopod were incorporated into allobasis, with 2 naked setae and 1 pinnate seta.

Maxilliped (Fig. 4D) comprising syncoxa, basis and 1-segmented endopod. Syncoxa with 2 plumose setae and several patches of spinules on syncoxa. Basis with 2 rows of spinules along palmar margin and row of spinules on outer lateral margin. Endopodal segment produced into strong pinnate claw.

**Figure 5.**
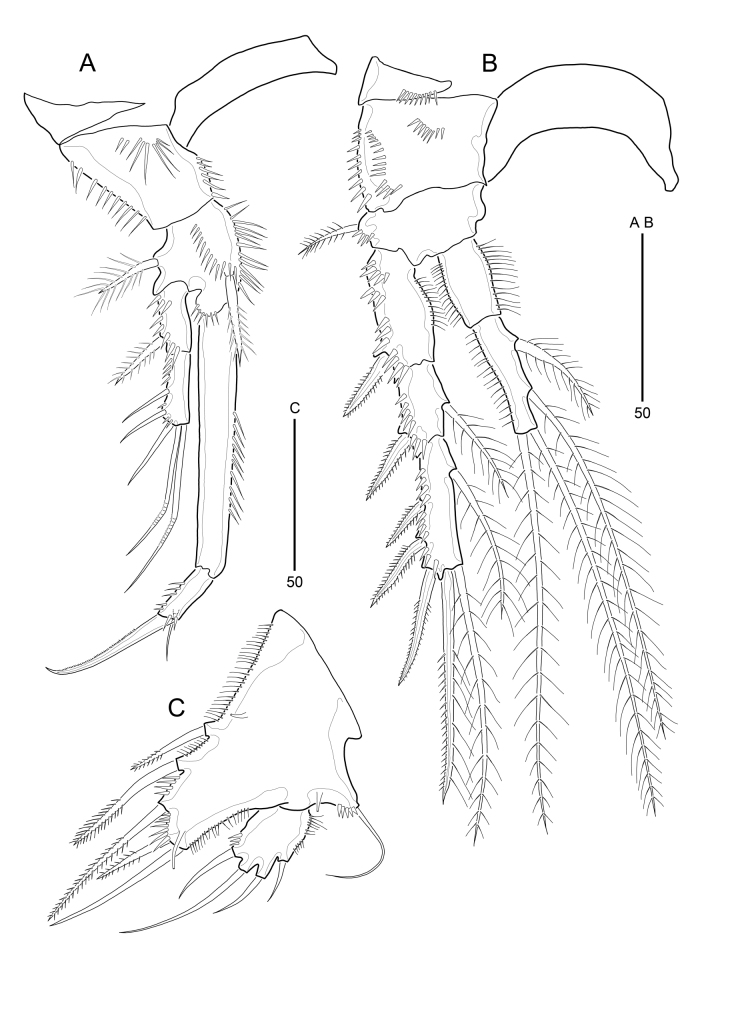
*Strictlaophontehupoensis* gen. nov., sp. nov., female; **A** P1; **B** P2; **C** P5.

**Figure 6.**
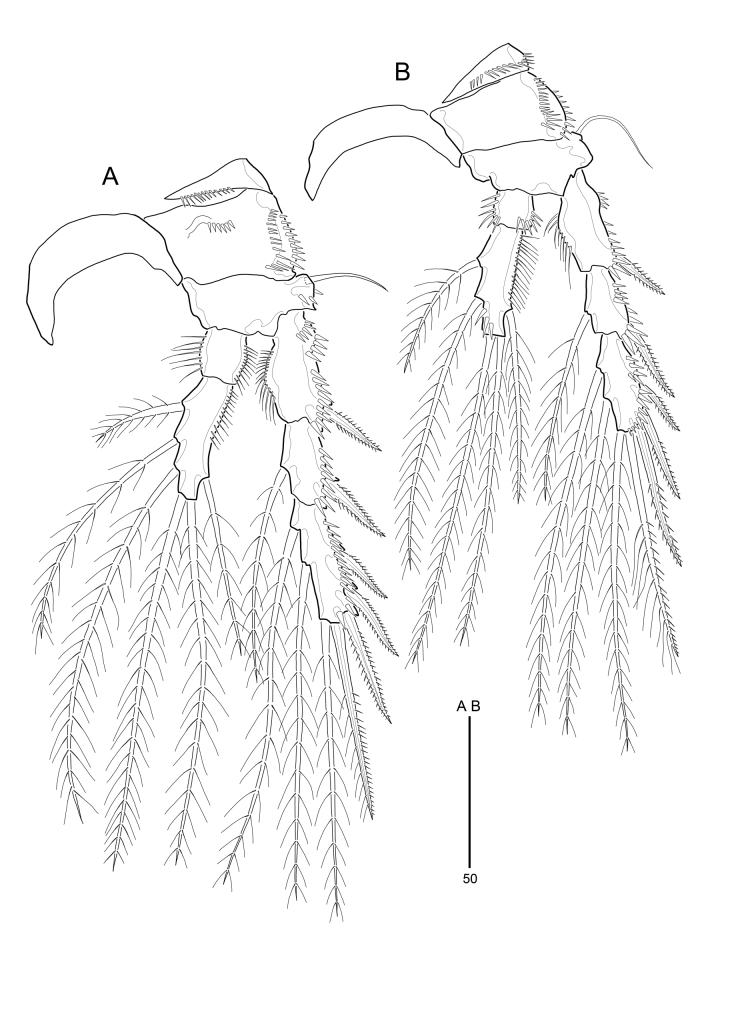
*Strictlaophontehupoensis* gen. nov., sp. nov., female; **A** P3; **B** P4.

Thoracic legs 1–4 (Fig. 5A, B and Fig. 6A, B) with wide naked intercoxal sclerite, triangle praecoxa, biramous, endopods 2-segmented, exopods 3-segmented, except for two-segmented endopod of P1. Coxae of P1-P4 larger than bases.

P1 (Fig. 5A). Coxa slightly longer than wide, with inner and outer spinular rows; with 1 spinular row on anterior surface. Basis with 1 plumose seta on outer margin and 1 strong pinnate seta near insertion of endopod; with spinular row along terminal margin and anterior surface and long spinules along inner margin. Exopod small, 2-segmented, not exceeding middle of Enp-1. Exp-1 with 1 pinnate outer spine. Exp-2 with 3 naked pinnate outer spines and 2 geniculate distal setae. Exopod with several spinules along outer margin. Endopod elongate and prehensile. Enp-1 2 times as long as exopod, with row of long spinules along inner margin. Enp-2 small, with 1 strong, denticulate claw apically and 1 small, naked seta subapically; with row of outer spinules and few anterior spinules.

P2–P4 (Fig. 5B and Fig. 6A, B). Praecoxa with row of distal spinules. Coxae with spinular rows along outer margin. Bases have a few spinules on anterior surface. Outer margin of basis with plumose seta (P2) or naked seta (P3-P4); each seta arising from short setophore. P2-P4 all endopod 2-segmented and exopod 3-segmented. All segments of exopod with elaborate pattern of spinules as illustrated; inner and outer margins of enp-1, outer margins of enp-2, inner margins of exp-1 with dense long slender spinules. P2-P4 enp-1 without inner and outer setae. P2 enp-2 1.4 times longer than enp-1; enp-2 with 2 plumose inner setae (1 long and 1 short) and 2 long plumose distal setae. P3 enp-2 2.3 times longer than enp-1; enp-2 with 3 plumose inner setae (2 long and 1 short), 1 plumose outer seta and 2 long plumose distal setae. P4 enp-2 2.5 times longer than enp-1; enp-2 with 2 plumose inner setae (1 long and 1 short), 1 plumose outer seta, and 2 long plumose distal setae. P2-P4 exp-2 shortest; exp-1 with 1 bipinnate outer spine; exp-2 with 1 plumose inner seta and 1 bipinnate outer spine. P2 exp-3 with 1 plumose inner seta, 3 bipinnate outer spine, and 2 long distal setae (1 plumose inner seta and 1 outer seta with both pinnate and plumose). P3-P4 exp-3 with 2 plumose inner seta, 3 bipinnate outer spine, and 2 long distal setae (1 plumose inner seta and 1 outer seta with both pinnate and plumose). Spine and setal formulae of P2-P4 as Table 1.

P5 (Fig. 5C) with separate exopod and baseoendopod, each with spinules as figured. Baseoendopod broad, forming outer setophore bearing basal naked seta; with 3 tube pores on anterior surface. Endopodal lobe exceeding distal margin of exopod; with 3 inner lateral pinnate setae and 1 pinnate seta and 1 naked seta on distal margin. Exopod small, slightly longer than wide, with 4 naked setae.

**Figure 7.**
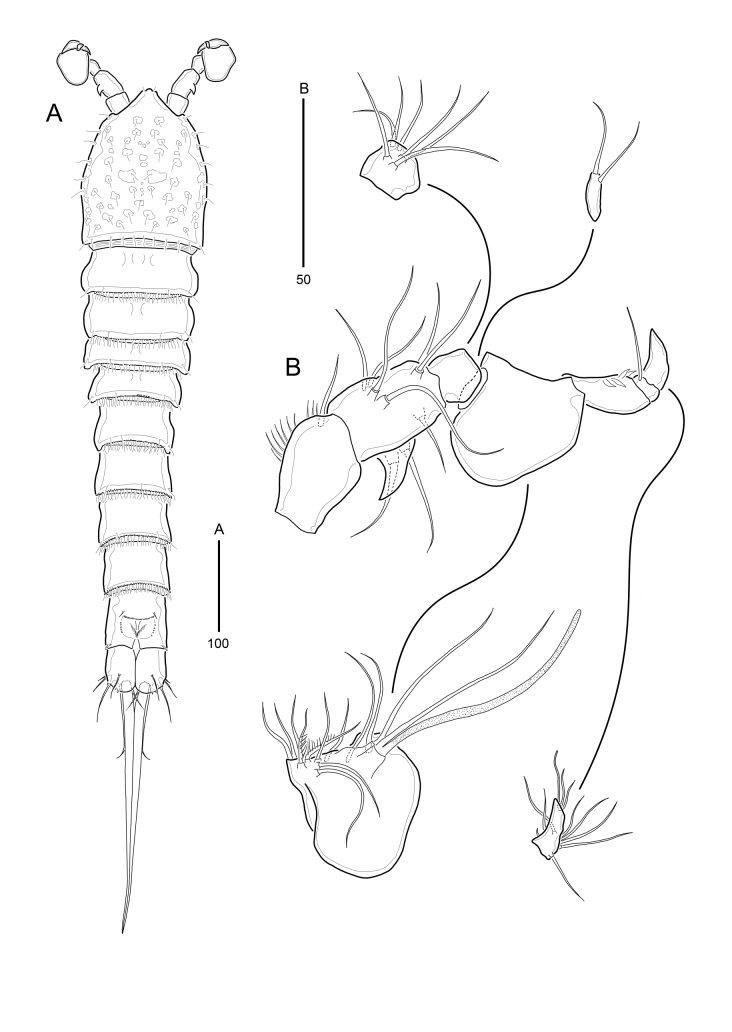
*Strictlaophontehupoensis* gen. nov., sp. nov., male; **A** habitus, dorsal; **B** antennule.

**Male**: Smaller and more slender than female. Total body length 965 μm (ranging from 894 - 1026 μm) measured from anterior margin of rostrum to posterior margin of caudal rami. Largest width measured at posterior margin of cephalic shield: 132 μm. Urosome distinctly narrower than prosome (Fig. 7A). All somites with densely spaced spinules on posterior margins. Sexual dimorphism in antennule, P3, P5, P6 and genital segmentation.

Urosome (Fig. 7A) comprised of P5-bearing somite, genital somite and 4 free abdominal somites.

Antennule 8-segmented (Fig. 7B), subchirocer, with geniculation between segments 5 and 6. Segment 1 with 1 row of long spinules along inner margin and with 1 naked seta on inner distal corner. Segment 2 with horn-shaped integumental protuberance on outer middle margin. Segment 4 represented by small sclerite. Segment 5 swollen. Segment 7 smallest. Segment 8 with shape of curved triangle. Armature formula: 1-[1], 2-[9], 3-[7], 4-[2], 5-[10 + 2 modified + (1 + ae)], 6-[4 modified], 7-[1], 8-[7 + 1 modified + acrothek]. Apical acrothek consisting of aesthetasc and 2 naked setae.

P2–P4. As in female, except for P3 with sexual dimorphism.

**Figure 8.**
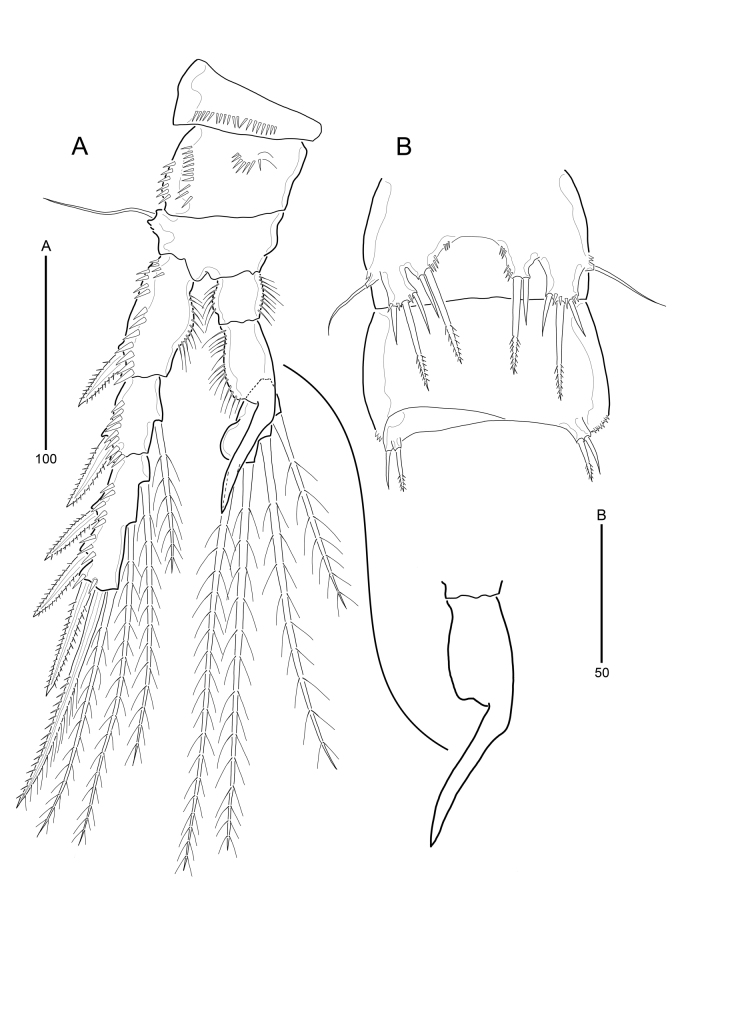
*Strictlaophontehupoensis* gen. nov., sp. nov., male; **A** P3; **B** P5, P6.

P3 (Fig. 8A). Endopod 3-segmented. Enp-2 with 1 strong inner apophysis, extending beyond enp-3; without spines and setae. Enp-3 with 2 plumose apical and 2 plumose inner setae. Exopod as in female.

P5 (Fig. 8B). Both legs fused medially. Baseoendopods fused to exopod, with setophore bearing outer naked basal seta. Endopodal and exopodal lobes vestigial, the former with one inner pinnate long seta and one outer naked shorter element, the latter with three elements of which the median longer and pinnate, the inner and outer shorter and naked.

P6 (Fig. 8B) asymmetrical, only one leg functional, the other fused to somite; each leg with two setae, of which inner element pinnate, outer element naked. Outer distal corner produced into short process bearing.

#### Etymology

The species name refers to the region where the new species was discovered, Hupo Port on the east coast of Korea.

## Discussion

The Laophontidae taxon is known for being very large, diverse and sometimes having heterogeneous characteristics within the family (Boxshall and Halsey 2004). Species belonging to this family live mainly in sandy subtidal areas in a wide variety of areas, from the tropics to the polar regions (Huys and Lee 2018). Recently, some genera and species of the Laophontidae family have been phylogenetically revised or reclassified and new genera and new species continue to be discovered. Most closely, in Korea, two new genera of *Parechinolaophonte* Song, Lee, Kim, George & Khim, 2023 and *Pseudechinolaophonte* Song, Lee, Kim, George & Khim, 2023 of this family were reported from Jeju Island in the South Sea of Korea by Song et al. (2023). In the eastern waters of Korea, where the specimens used in this study were collected, *Philippiphonte* Huys & J. Lee, 2018, a new genus of the Laophontidae family was recently described from Dokdo Island in the East Sea (Huys and Lee 2018).

The specimens analysed in our study exhibit all of the major characteristics generally shared by the Laophontidae family listed later. This family has a cylindrical body shape, reduced one-segment antennary exopod with 1–4 setae, P1 enp-1 without inner seta, P1 enp-2 with claw and 1 small accessory seta, p5 with a distinct exopod that is not fused at the basis and developed sexual dimorphism (Huys et al. 1996, Wells 2007).

According to Well’s Key (Wells 2007), the character traits of this undescribed specimen of the Laophontidae family do not match any existing genus. This laophontid genus has unique morphological characteristics that distinguish it from other genera. This new genus, *Strictlaophonte* has a very rare combination in its P5, with an endopod bearing 5 setae and an exopod smaller than the endopod, having only 4 setae. In addition, the new genus has P1 with 2-segmented exopod, enp-1 of P2-P4 without setae, and a very unique shape of the caudal rami and terminal setae.

*Strictlaophonte* gen. nov. is closely related to the genera *Bathylaophonte* Lee W. & Huys, 1999 (see Lee and Huys (1999)) and *Marbefia* Huys & Lee, 2009 (see Huys and Lee (2009)). In particular, *Bathylaophonte* is completely consistent with *Strictlaophonte* in terms of spine and setal formulae of the endopod and exopod of P2-P4. On the other hand, *Marbefia* Huys & Lee, 2009 showed that, although the P2-P4 armature formula is mostly identical, the endopods of P3 and P4 have different numbers of setae. In addition, *Bathylaophonte* Lee W. & Huys, 1999 and *Marbefia* Huys & Lee, 2009 showed that the number of antennule segments in males and females (7 and 8, respectively) were the same as in *Strictlaophonte* gen. nov. However, *Strictlaophonte* gen. nov. is crucially different from the above two genera in that the P5 exopod has only four setae and the P1 exopod has two segments. The number of segments and setae in the P1-P5 is a particularly inportant chracteristic in determining the laophontid genera.

The sexual dimorphism of the thoracic leg in the family Laophontidae is more frequent than that in any other family. Wells (2007) reported that the P2–P4 setation is variable in Laophontidae, with morphological variation often occurring within a population between the sexes. In *Strictlaophonte* gen. nov., sexual dimorphism also occurs in the second endopod of male P3. The distal margin of the second endopod has a long and strong apophysis that extends beyond the length of the third endopod. In connection with this, the endopod of male P3 has three segments, differing from that of the female with two segments.

*Strictlaophonte* gen. nov. is characterised by closely-attached caudal rami, as well as terminal V setae that are tightly attached parallel to each other. The proximity of the caudal rami is mainly observed in planktonic copepods, such as the order Cyclopoida. This morphological feature is rarely seen in benthic harpacticoids, but some genera of the Aegisthidae family, which live in the deep sea and have completely different body shapes, shows similar morphology (Por 1969, Conroy-Dalton and Huys 1999). Nevertheless, this characteristic has not yet been reported in the Laophontidae family and is the unique difference distinguishing this new genus from other genera.

## Supplementary Material

XML Treatment for Strictlaophonte

XML Treatment for Strictlaophonte hupoensis

## Figures and Tables

**Table 1.** *Strictlaophonte* gen. nov., P2-P4 armature formula.

	**Exopod**	**Endopod**
P2	0.1.123	0.220
P3	0.1.223	0.321 [0.0.220 in ♂]
P4	0.1.223	0.221
